# Faux kyste du pancréas drainé spontanément dans le colon

**DOI:** 10.11604/pamj.2013.15.81.3005

**Published:** 2013-06-30

**Authors:** Karim Ibn Majdoub Hassani, Khalid Mazaz

**Affiliations:** 1 Faculté de médecine et de pharmacie de Fès, Université Sidi Mohammed Ben Abdellah, département de Chirurgie, CHU Hassan II Fès, BP: 1893; Km2.200, Route de Sidi Hrazem, Fès 30000, Maroc

**Keywords:** Faux kyste, pancréas, pancréatite aigue, pseudocyst, pancréas, acute pancreatitis

## Image en médecine

Les faux kystes du pancréas (FKP) se développent chez environ 2% des patients présentant une pancréatite aigüe. Ils peuvent évoluer vers la régression spontanée ou se compliquer d'une hémorragie, de compression ou de rupture. Le drainage spontané des FKP dans un viscère creux (duodénum, estomac, ou colon) reste un mode d’évolution exceptionnel. Nous rapportant le cas d'une patiente âgée de 74 ans, cholécystectomisée il y a 14 ans, qui a présenté une pancréatite stade (E) de Balthazar après une sphinctérotomie endoscopique pour lithiase résiduelle de la voie biliaire principale. L’évolution était favorable sous traitement symptomatique avec une nette amélioration. Un mois plus tard la patiente consulte pour des douleurs abdominales avec un syndrome infectieux et une masse épigastrique sensible à l'examen abdominal. La tomodensitométrie (TDM) abdominale objective la présence d'un FKP communiquant largement avec le colon transverse. On décide alors de surveiller la malade sous traitement antibiotique pendant 3 semaines, l’évolution est favorable avec disparition complète de la symptomatologie. La TDM de contrôle un mois plus tard montre la régression complète du FKP.

**Figure 1:**
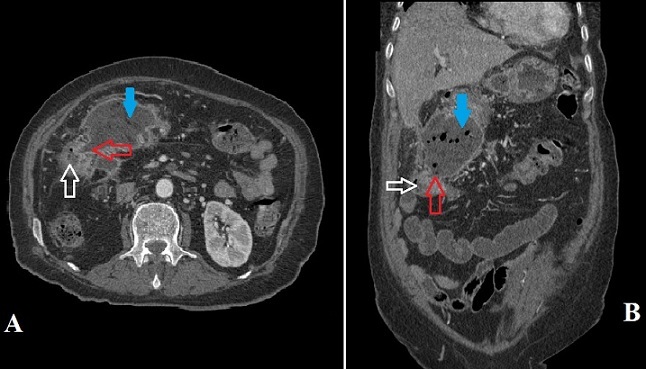
Images scannographiques (A) coupe axiale, (B) coupe coronal montrant la communication (flèche rouge) du FKP (flèche bleue) avec le côlon droit (flèche blanche)

